# Air-liquid interface culture promotes maturation and allows environmental exposure of pluripotent stem cell–derived alveolar epithelium

**DOI:** 10.1172/jci.insight.155589

**Published:** 2022-03-22

**Authors:** Kristine M. Abo, Julio Sainz de Aja, Jonathan Lindstrom-Vautrin, Konstantinos-Dionysios Alysandratos, Alexsia Richards, Carolina Garcia-de-Alba, Jessie Huang, Olivia T. Hix, Rhiannon B. Werder, Esther Bullitt, Anne Hinds, Isaac Falconer, Carlos Villacorta-Martin, Rudolf Jaenisch, Carla F. Kim, Darrell N. Kotton, Andrew A. Wilson

**Affiliations:** 1 Center for Regenerative Medicine of Boston University and Boston Medical Center, Boston, Massachusetts, USA.; 2 The Pulmonary Center and Department of Medicine, Boston University School of Medicine, Boston, Massachusetts, USA.; 3 Stem Cell Program and Divisions of Hematology/Oncology and Pulmonary & Respiratory Diseases, Boston Children’s Hospital, Boston, Massachusetts, USA.; 4 Harvard Stem Cell Institute, Cambridge, Massachusetts, USA.; 5 Department of Genetics, Harvard Medical School, Boston, Massachusetts, USA.; 6 Whitehead Institute for Biomedical Research, Cambridge, Massachusetts, USA.; 7 Department of Physiology & Biophysics, Boston University, Boston, Massachusetts, USA.; 8 Boston University School of Medicine, Boston, Massachusetts, USA.; 9 Department of Biology, Massachusetts Institute of Technology, Cambridge, Massachusetts, USA.

**Keywords:** Stem cells, Human stem cells, iPS cells

## Abstract

Type 2 alveolar epithelial cells (AT2s), facultative progenitor cells of the lung alveolus, play a vital role in the biology of the distal lung. In vitro model systems that incorporate human cells, recapitulate the biology of primary AT2s, and interface with the outside environment could serve as useful tools to elucidate functional characteristics of AT2s in homeostasis and disease. We and others recently adapted human induced pluripotent stem cell–derived AT2s (iAT2s) for air-liquid interface (ALI) culture. Here, we comprehensively characterize the effects of ALI culture on iAT2s and benchmark their transcriptional profile relative to both freshly sorted and cultured primary human fetal and adult AT2s. We find that iAT2s cultured at ALI maintain an AT2 phenotype while upregulating expression of transcripts associated with AT2 maturation. We then leverage this platform to assay the effects of exposure to clinically significant, inhaled toxicants including cigarette smoke and electronic cigarette vapor.

## Introduction

Type 2 alveolar epithelial cells (AT2s) reside in the distal lung, where they function as facultative progenitor cells of the alveolus, synthesize and secrete surfactant, and respond to inhaled immune stimuli (1). Their central role in the biology of this lung compartment makes the ability to model AT2 homeostatic or aberrant function in vitro a desirable goal for lung researchers. In vitro models of human epithelia in air-liquid interface (ALI) culture have served as useful platforms that promote the differentiation and maturation of epithelial cells and permit in vitro modeling of infections and environmental exposures (2–10). Primary human AT2s can be cultured and passaged in vitro as 3-dimensional (3D) organoids (11, 12) but are sourced from explant or donor lungs and tend to lose expression of AT2-specific surfactants when cultured in 2D monolayers (13). Human induced pluripotent stem cells (iPSCs) can be directed to differentiate to AT2s (iAT2s) in 3D spheres, where they transcriptomically resemble primary human AT2s (14–16) and recapitulate disease-specific phenotypes of the human subject of origin (14, 17). We and others recently reported the successful adaptation of iAT2s to ALI culture and the application of that system for disease modeling (18–20). Here, we provide in-depth characterization of iAT2s in ALI culture in comparison with fetal and adult primary AT2s. We transcriptomically profile iAT2s and find that, when cultured at ALI, they upregulate key markers of AT2 maturation as they downregulate cell cycle–associated transcripts relative to those cultured in 3D spheres. Finally, we demonstrate that iAT2s cultured at ALI respond to clinically significant, injurious environmental stimuli including cigarette smoke and electronic cigarette vapor.

## Results

### Human iAT2s maintain AT2-like identity in ALI cultures.

**Figure 1:**
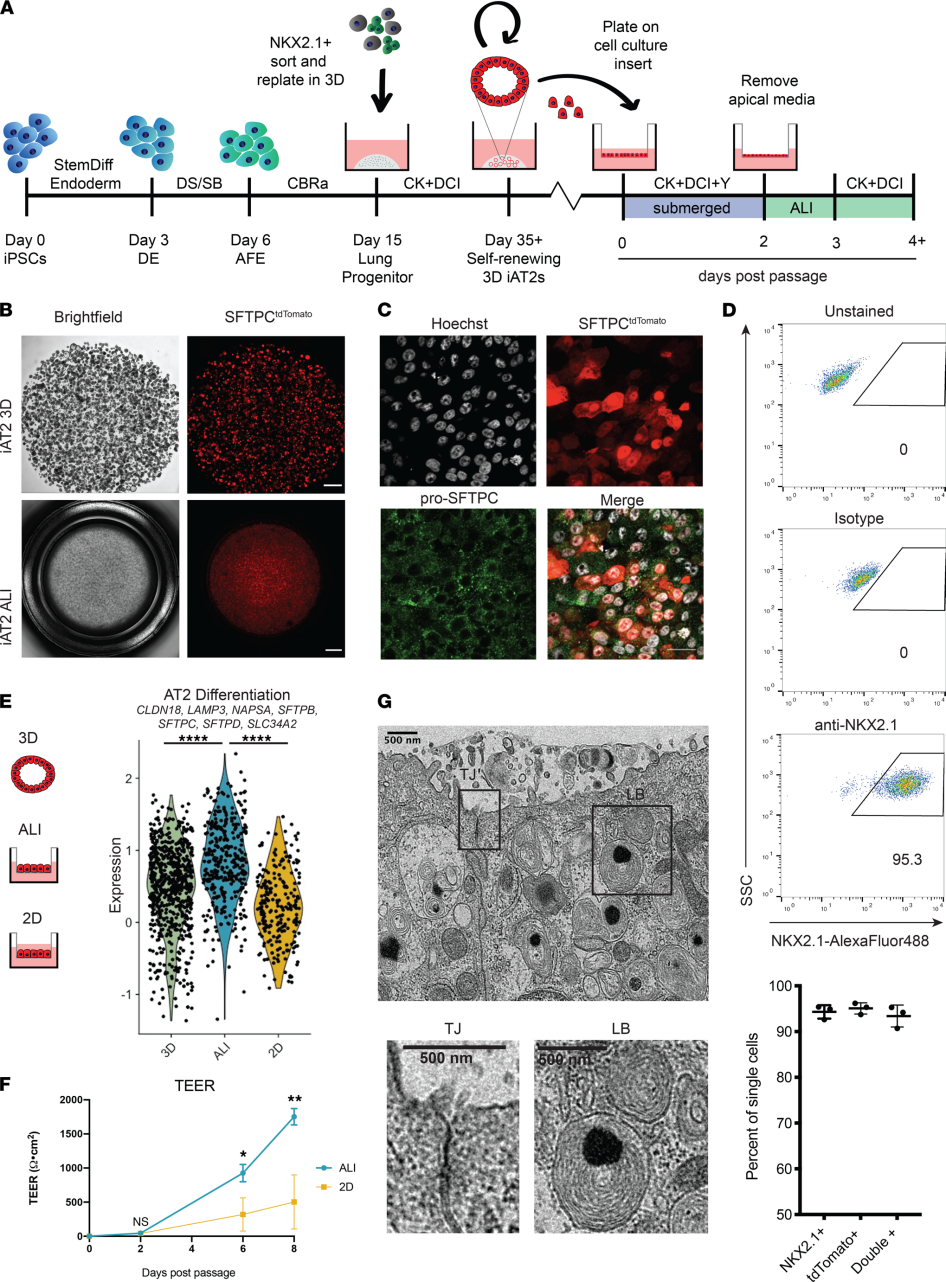
iAT2s in ALI culture express AT2 transcripts, tight junctions, and lamellar bodies. (**A**) iAT2 differentiation and ALI culture schematic. iAT2s cultured as 3D spheres were dissociated and plated on cell culture inserts to generate ALI cultures. Apical medium was removed after 2 days in submerged culture. DE, definitive endoderm; AFE, anterior foregut endoderm. DS/SB = 2 μM dorsomorphin + 10 μM SB431542; CBRa = 3 μM CHIR99021 + 10 ng/mL BMP4 + 100 nM retinoic acid; CK = 3 μM CHIR99021 + 10 ng/mL KGF; DCI = 50 nM dexamethasone + 0.1 mM cAMP + 0.1 mM IBMX; and Y = 10 μM Rho-associated kinase inhibitor (Y-27632). (**B**) Bright-field and fluorescence imaging of iAT2s targeted with a tdTomato-encoding cassette to the endogenous SFTPC locus cultured in 3D and at ALI for 10 days. Scale bars: 1 mm. (**C**) Immunofluorescence images of pro-SFTPC (green) and SFTPC-tdTomato (red) in iAT2s cultured at ALI for 10 days. Scale bar: 25 µm. (**D**) Representative flow cytometry plots of NKX2.1 expression in iAT2 ALI cultures and quantification of NKX2.1 and SFTPC-tdTomato expression (*n* = 3; additional flow cytometry plots in Supplemental Figure 1). (**E**) Violin plot of AT2 differentiation module (*CLDN18*, *LAMP3*, *NAPSA*, *SFTPB*, *SFTPC*, *SFTPD*, and *SLC34A2*) score in iAT2s cultured in 3D, ALI, or 2D submerged conditions on day 10 after passage by scRNA-Seq (1-way ANOVA). (**F**) Transepithelial electrical resistance of iAT2 ALI or 2D submerged cultures 0–8 days after passage (unpaired 2-tailed Student’s t test, *n* = 3 per condition). (**G**) Electron micrograph of iAT2 ALI culture showing a tight junction (TJ) and lamellar bodies (LB). Scale bars : 500 nm. Data are shown as mean ± SD. *P ≤ 0.05; **P ≤ 0.01; ****P ≤ 0.0001.

To develop an ALI culture system for human alveolar epithelial cells, we generated iAT2s (14, 21) from a human iPSC line carrying a tdTomato-encoding cassette targeted to the *SFTPC* locus (SPC2; refs. 15, 22). We established 3D cultures of self-renewing iAT2 spheres that could be serially passaged to provide a stable source of human AT2-like cells (18, 22). We then dissociated 3D iAT2s and plated single-cell iAT2s on cell culture inserts in submerged culture. After 2 days of culture in these conditions, we aspirated medium from the apical chamber to establish ALI cultures (Figure 1A).

We first asked whether ALI culture altered the cellular identity of iAT2s. To do so, we measured transcript expression levels of the key lung epithelial transcription factor *NKX2-1* and the canonical AT2 marker *SFTPC* over time. iAT2s expressed *NKX2-1* and *SFTPC* at similar levels in 3D and ALI culture up to 10 days after passage based on quantitative PCR (qPCR) (Supplemental Figure 1A; supplemental material available online with this article; https://doi.org/10.1172/jci.insight.155589DS1). Active expression from the *SFTPC* locus was maintained in iAT2s cultured at ALI as indicated by *SFTPC*-driven expression of the tdTomato reporter (Figure 1B), as well as by expression of pro-SFTPC protein observed by immunofluorescence staining (Figure 1C). Quantification by flow cytometry demonstrated that iAT2s cultured at ALI expressed NKX2-1 protein (94% ± 1.5%) and SFTPC^tdTomato^ (95% ± 1.2%), with 93% positive for both markers (93% ± 2.4%) (Figure 1D and Supplemental Figure 1B).

To define the global transcriptomic effects of our culture conditions, we analyzed iAT2s cultured in parallel either as 3D spheres, at ALI, or on cell culture inserts maintained in submerged conditions (“2D”) by single-cell RNA-Seq (scRNA-Seq) 10 days after passage (Supplemental Figure 1, C–F). We found that iAT2s in ALI culture, compared with either 3D or 2D culture, significantly upregulated a set of genes associated with the in vivo differentiation of human AT2s (15): *CLDN18*, *LAMP3*, *NAPSA*, *SFTPB*, *SFTPC*, *SFTPD,* and *SLC34A2* (Figure 1E and Supplemental Figure 2). Similar to 3D conditions, ALI culture of iAT2s did not upregulate expression of genes associated with alveolar type 1 cells (AT1; *CTSE*, *CAV1*, *AGER*) or genes associated with epithelial-mesenchymal transition (EMT) (*SNAI1* and *COL1A1*; *TWIST1* not detected) (Supplemental Figure 2).

We next looked at the effects of ALI culture on iAT2 barrier function and found that ALI-cultured iAT2s formed an intact epithelium, reaching transepithelial electrical resistance (TEER) of 1753 ± 120 Ω·cm^2^ by 8 days after passage (*n* = 3 per condition) (Figure 1F), significantly greater than that observed in 2D submerged cultures at the same time point (503 ± 397 Ω·cm^2^) (Figure 1F). Consistent with this observation, ultrastructural analysis of ALI cultures by transmission electron microscopy (TEM) revealed the presence of tight junctions together with lamellar bodies, specialized surfactant-containing organelles unique among lung cell types to AT2s (Figure 1G).

### iAT2s mature in ALI cultures in association with decreased cell cycling.

**Figure 2:**
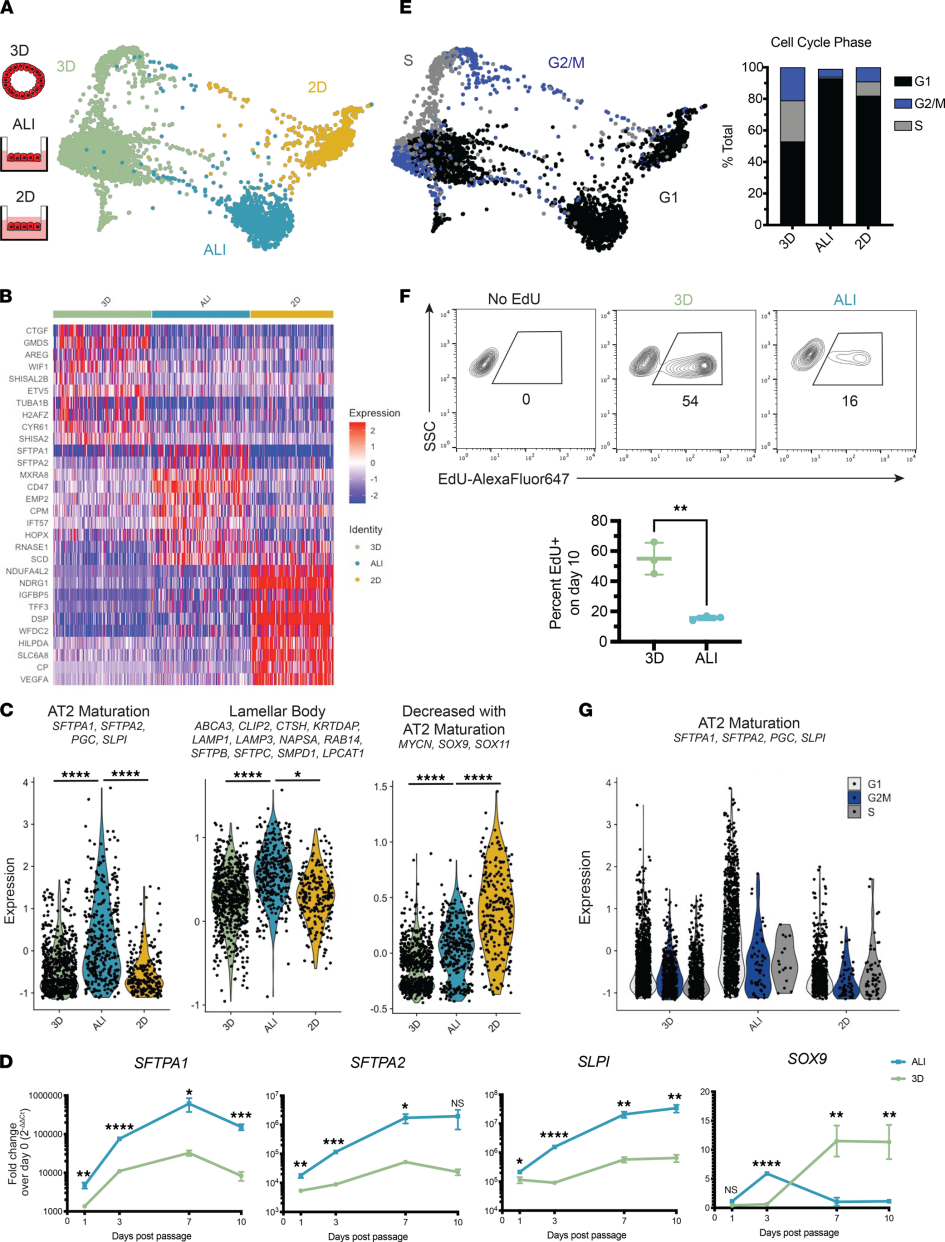
iAT2 maturation in ALI culture is associated with decreased cell cycling. (**A**) SPRING plot of iAT2s cultured in 3D, ALI, or 2D profiled by scRNA-Seq on day 10 after passage, colored by cell culture format. (**B**) Heatmap of top 10 differentially expressed genes per sample. (**C**) Violin plots of module scores for AT2 maturation genes, lamellar body–associated genes, and genes that are decreased with AT2 maturation (1-way ANOVA). (**D**) qPCR of *SFTPA1*, *SFTPA2*, *SLPI*, and *SOX9* at 1, 3, 7, and 10 days after passage in iAT2s cultured at 3D or ALI (unpaired 2-tailed Student’s *t* test*, n* = 3). (**E**) SPRING plot of iAT2s cultured in 3D, ALI, and 2D profiled on day 10 after passage by scRNA-Seq, colored by inferred cell cycle phase; quantification of inferred cell cycle phase by cell culture format. (**F**) Flow cytometry plots of iAT2s exposed to a 24-hour pulse of EdU and collected on day 10 after passage (unpaired 2-tailed Student’s *t* test*, n* = 3). (**G**) Violin plots of AT2 maturation gene module score by cell cycle phase and cell culture format. Data are shown as mean ± SD. *P ≤ 0.05; **P ≤ 0.01; ***P ≤ 0.001; ****P ≤ 0.0001.

ALI culture has been previously noted to promote differentiation of epithelial cells in the lung airway and other tissues (2–10). Further analysis of scRNA-Seq data provided additional insight into the effects of ALI culture on iAT2s (Figure 2A). We observed that top genes upregulated at ALI relative to other conditions included genes encoding surfactant proteins (*SFTPA1*, *SFTPA2*), as well as genes associated with lung progenitor cells (*CD47, CPM*) (16, 23) and *HOPX,* which is expressed by human AT2s (Figure 2B). iAT2s cultured at ALI likewise had higher expression levels of gene sets associated with AT2 maturation (15) and with alveolar epithelial lamellar bodies compared with other conditions (Figure 2C). In contrast, genes that are downregulated in the course of primary AT2 maturation (15) — *MYCN*, *SOX9*, and *SOX11* — were most highly expressed in 2D submerged conditions (Figure 2C). Using qPCR, we confirmed upregulation of *SFTPA1*, *SFTPA2*, and *SLPI* with concurrent downregulation of *SOX9* in iAT2s cultured in ALI compared with 3D (Figure 2D and Supplemental Figure 3). We observed a peak in expression of genes associated with AT2 maturation and nadir in *SOX9* expression at 7–10 days after passage (Figure 2D).

We next evaluated expression of transcripts associated with cell cycle phase to infer the cell cycle state of iAT2s in different culture conditions. Only 6% of iAT2s at ALI expressed transcripts associated with active cell cycling (S, G_2_, or M phases), compared with 47% of iAT2s in 3D culture and 18% of those in 2D submerged conditions (Figure 2E). Decreased cell cycling at ALI compared with 3D was further confirmed by EdU flow cytometry assay: following a 24-hour pulse of EdU on day 10 after passage, 16% ± 2% of iAT2s at ALI had incorporated EdU, compared with 55% ± 11% of those in 3D culture (Figure 2F). We next asked whether iAT2 maturation and decreased cell cycling were independently associated with culture at ALI. We found that, across culture conditions, cells that were not in S/G_2_/M phases expressed significantly higher levels of the gene set associated with AT2 maturation based on scRNA-Seq and that these genes were most highly expressed in noncycling iAT2s at ALI (Figure 2G). Taken together, these results indicate that culturing iAT2s in ALI results in a transition from a proliferative state to a more mature cell state.

### iAT2s resemble primary AT2s in epithelial gene expression.

We have previously compared RUES2-derived iAT2s cultured as 3D spheres on day 35 of differentiation and sorted on SFTPC^tdTomato^ (day 35) with undifferentiated human pluripotent stem cells (hPSCs, day 0), hPSC-derived NKX2.1^+^ lung progenitors after 15 days of directed differentiation (day 15), distal epithelial cells from human fetal lungs (HFLs) at 16–17.5 weeks of gestation (early HFL) and 20–21 weeks (late HFL), and adult HTII-280–sorted (24) primary AT2 controls (adult sorted AT2) by bulk RNA-Seq (*n* = 3 per group) (14, 15).

In order to delineate the effects of culture conditions on both primary AT2s and iAT2s in the context of this previously generated data set, we sorted AT2s on HTII-280 (24) from 2 additional human donor lungs, one 32-year-old adult donor and one 13-month-old pediatric donor, and cultured them in vitro for 106 days in triplicate before collecting RNA. RNA from iAT2s cultured in 3D spheres or ALI after 281 total days of differentiation was collected on day 10 after passage. We transcriptomically profiled these 4 additional sample groups (adult cultured AT2, pediatric cultured AT2, iAT2 3D, and iAT2 ALI) by RNA-Seq. Since primary AT2s were cultured in the presence of MRC5 fibroblasts, a scRNA-Seq data set of an independent primary AT2 in vitro sample was utilized to generate a signature matrix to deconvolve AT2 and fibroblast transcript contributions (Supplemental Table 1). We merged imputed primary cultured AT2 expression matrices with newly generated iAT2 3D and ALI, as well as previously generated day 0, day 15, day 35, early and late HFL, and adult sorted AT2 expression matrices.

**Figure 3:**
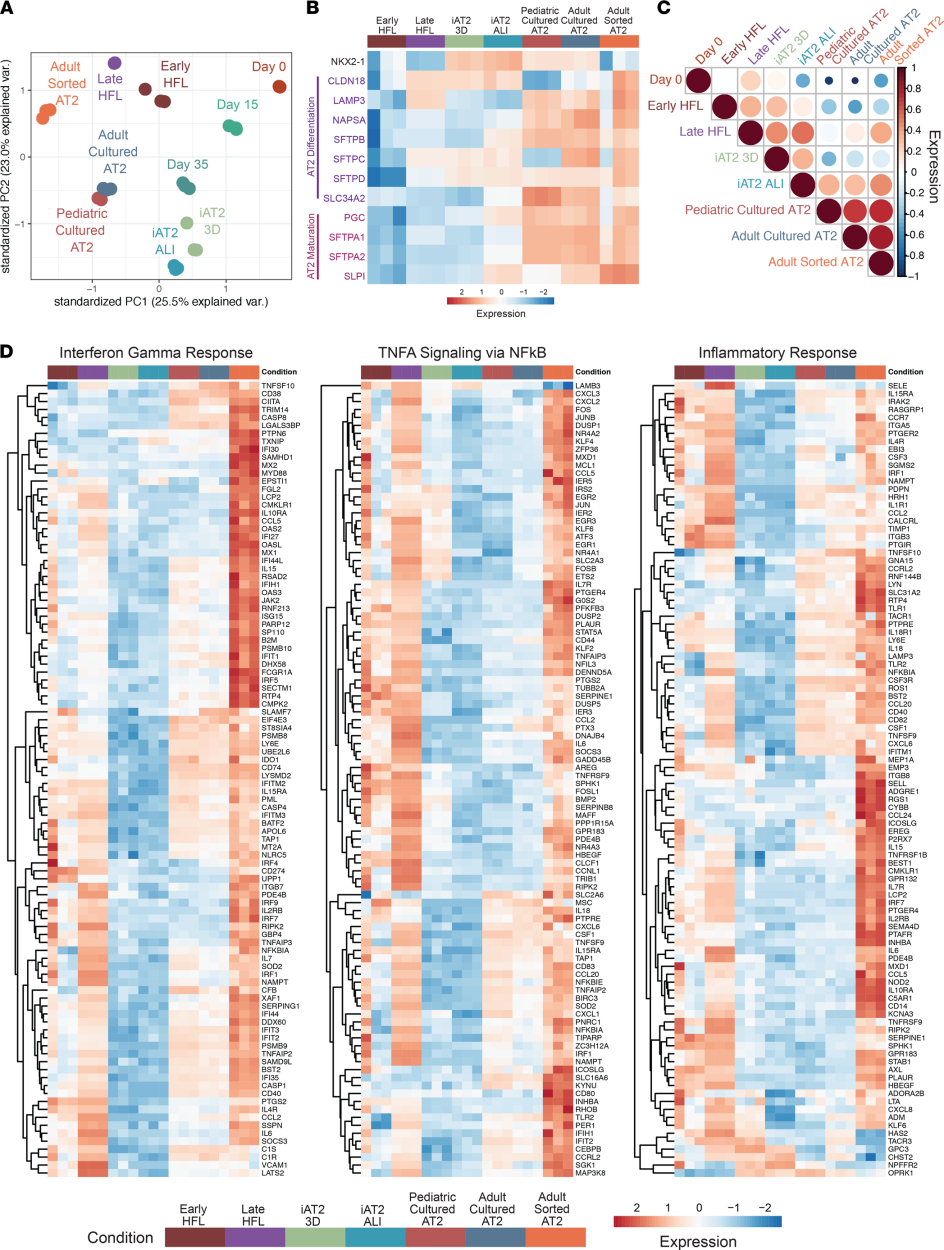
iAT2s resemble primary AT2s in epithelial gene expression and differ in immune response state. (**A**) Principal component analysis (PCA) of 10 samples (*n* = 3 per condition) showing global transcriptomic variance (%) of PC1 and PC2. Day 0, undifferentiated PSCs; day 15, day 15 *NKX2.1*^+^ progenitors; day 35 = day 35 tdTomato^+^ iAT2s; iAT2 3D, day 281 iAT2s, 10 days after passage in self-renewing 3D culture; iAT2 ALI, day 281 iAT2s, 10 days after passage to cell culture inserts; early HFL, weeks 16–17.5 (early canalicular) human fetal lung; late HFL, week 20–21 (late canalicular) human fetal lung; adult sorted AT2s, adult HTII-280–sorted AT2s; adult cultured AT2, adult HTII-280–sorted AT2s cultured in vitro; pediatric cultured AT2, pediatric HTII-280–sorted AT2s cultured in vitro. (**B**) Heatmap of row-normalized expression of key AT2 markers across iAT2 culture formats and primary lung samples. (**C**) Heatmap of Pearson correlation coefficients calculated between each sample based on normalized expression of human lung epithelial genes from an independent data set (25) and plotted as an average across samples for each group (see Supplemental Figure 4 for replicate values). (**D**) Heatmaps of row-normalized *Z* scores of top 100 differentially expressed genes between adult sorted AT2 versus iAT2 ALI (FDR < 0.05, ranked by log fold change) in the Hallmark gene sets Interferon Gamma Response, TNFA Signaling via NFkB, and Inflammatory Response.

By principal component analysis (PCA), we found that replicates from each sample clustered together and that PSC-derived samples segregated from primary samples along principal component 1 (PC1) (Figure 3A). PSC-derived samples segregated by day of differentiation along PC2, with day 281 3D and ALI iAT2s also clustering independently from each other (Figure 3A). Adult freshly sorted AT2s clustered independently from either adult or pediatric AT2s cultured in CK+DCI medium (see Methods) (Figure 3A). Profiling of expression of genes associated with AT2 maturation (15) demonstrated that their expression in iAT2s is greater in ALI culture than in 3D spheres (Figure 3B).

To quantify the extent to which iAT2s at ALI resemble primary AT2s transcriptomically, we calculated Pearson correlation coefficients between each sample based on normalized expression of human lung epithelial genes from an independent data set (25). We found that iAT2s more closely resemble adult AT2s when cultured in ALI (*r* = 0.67 ± 0.08) than when cultured as 3D spheres (*r* = 0.44 ± 0.04) in terms of their human lung epithelial gene expression (Figure 3C, Supplemental Figure 4, and Supplemental Table 3).

### iAT2s at ALI differ from primary sorted AT2s in an immune response state.

Next, we asked what biological processes or states differ between primary adult sorted AT2s and iAT2s cultured at ALI. Gene set enrichment analysis (GSEA) of adult sorted AT2s and iAT2 ALI revealed that the top differentially expressed gene sets in primary AT2s involved upregulation of immune pathways (FDR < 0.05; Supplemental Table 4). These top gene sets include the viral response processes Interferon Gamma Response and Interferon Alpha Response, as well as gene sets that reflect inflammatory states such as TNFA Signaling via NFkB, Inflammatory Response, and IL6 JAK STAT3 Signaling (FDR < 0.05; Supplemental Table 4 and Figure 3D). Heatmaps of normalized expression of the top 100 differentially expressed genes within these gene sets demonstrate that the members of the gene sets Interferon Gamma Response, TNFA Signaling via NFkB, and Inflammatory Response are increased in all primary and primary cultured samples compared with iPSC-derived samples, with markedly higher expression in adult sorted AT2s and HFL samples compared with all other samples (Figure 3D).

### iAT2s respond to combustible cigarette smoke and electronic cigarette vapor.

**Figure 4:**
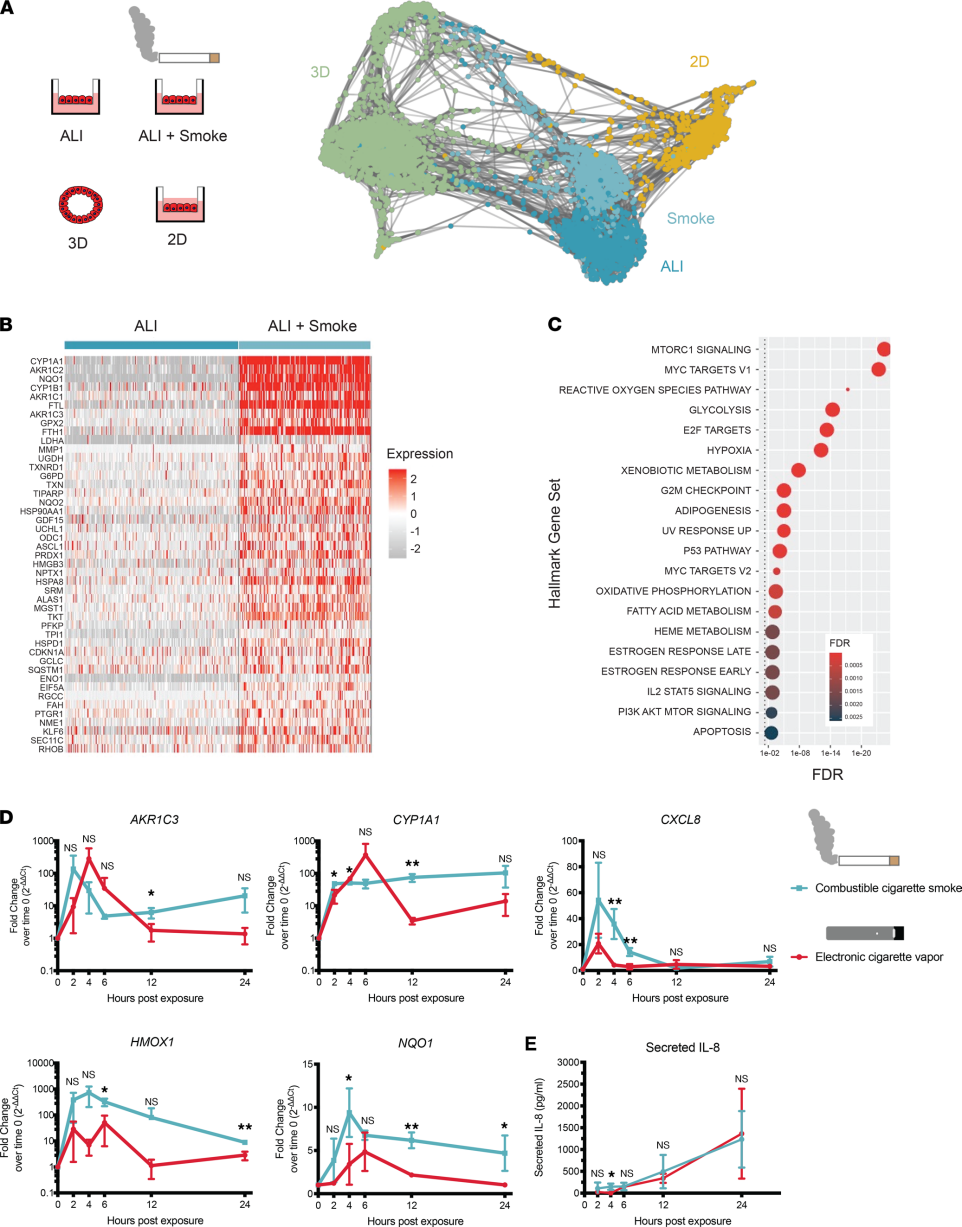
iAT2s respond to combustible cigarette smoke and electronic cigarette vapor. (**A**) SPRING plot of iAT2s cultured in 3D, 2D, or ALI and profiled by scRNA-Seq 12 hours after cigarette smoke exposure (Smoke) or air exposure (ALI), colored by condition. (**B**) Heatmap of top differentially expressed genes between cigarette smoke–exposed and air-exposed iAT2s in ALI culture. (**C**) Gene set enrichment analysis (GSEA, hypeR using Hallmark gene sets) of top upregulated genes in cigarette smoke–exposed iAT2s compared with air-exposed iAT2s (dotted line represents statistical significance threshold; FDR < 0.05). (**D**) qPCR of *AKR1C3*, *CYP1A1*, *CXCL8*, *HMOX1*, and *NQO1* in iAT2s at 0, 2, 4, 6, 12, and 24 hours after exposure to combustible cigarette smoke or electronic cigarette vapor (unpaired 2-tailed Student’s *t* test, *n* = 3). (**E**) ELISA for IL-8 secreted by iAT2s into basolateral medium by 0, 2, 4, 6, 12, and 24 hours after exposure to combustible cigarette smoke or electronic cigarette vapor (unpaired 2-tailed Student’s t test, *n* = 3). Data are shown as mean ± SD. *P ≤ 0.05; **P ≤ 0.01.

Since they recapitulate the air-interface that exists in the lung alveolus in vivo, ALI culture conditions might be applied to understand the alveolar epithelial response to inhaled factors. Cigarette smoke is a common, noxious exposure known to lead to diseases of both the airway and alveoli, but it has been challenging to model in human alveolar cells. We therefore exposed iAT2 ALI cultures to gas-phase cigarette smoke (5% smoke by volume, diluted in humidified room air) or humidified room air (negative control) using an in vitro smoke exposure instrument before transcriptomically profiling them by scRNA-Seq 12 hours later (Figure 4A). Cigarette smoke–exposed iAT2s cluster independently from controls (Figure 4A) and strongly upregulate several canonical smoke-response genes, including the extrahepatic cytochrome P450 enzymes *CYP1A1* and *CYP1B1*; members of the aldo-keto reductase (AKR) family *AKR1C2*, *AKR1C1*, and *AKR1C3*; the target of master antioxidant regulator of NRF2 *NQO1*; and the ferritin subunits *FTL* and *FTH1* (Figure 4B). GSEA of upregulated smoke-responsive genes revealed enrichment in Hallmark gene sets including the Reactive Oxygen Species Pathway, Hypoxia, and Xenobiotic Metabolism (FDR < 0.05; Figure 4C). 

The use of emerging tobacco products such as electronic nicotine delivery systems (ENDS), or electronic cigarettes, likewise results in exposure of alveolar cells to a complex mixture of volatile compounds with poorly understood consequences. To characterize the cell-intrinsic kinetic response to electronic cigarette vapor in comparison with that of cigarette smoke, we exposed iAT2s to cigarette smoke, electronic cigarette vapor, or air (5% smoke or vapor by volume, diluted in humidified room air) before collecting RNA as well as basolateral medium at time points up to 24 hours after exposure. We found that electronic cigarette vapor exposure induced expression of a set of cigarette smoke–responsive genes (*AKR1C3*, *CYP1A1*, *CXCL8*, *HMOX1*, and *NQO1*) at levels and with a kinetic similar to that induced by cigarette smoke, peaking transcriptionally 2–6 hours after exposure (Figure 4D). Finally, we observed that iAT2s secrete IL-8, encoded by *CXCL8*, at similar kinetics for 24 hours following exposure to either electronic cigarette vapor or cigarette smoke exposure (Figure 4, D and E).

## Discussion

Our results demonstrate that ALI culture of human iPSC–derived AT2s promotes their maturation while reducing their proliferation and enables the study of their response to environmental exposures such as cigarette smoke and electronic cigarette vapor. iAT2s can be serially passaged indefinitely in 3D culture and, thus, represent a shareable and stable resource (14, 15, 18) that can be used to generate ALI cultures with utility for modeling the cellular response to airborne stimuli. 

Primary human AT2s can lose their AT2-like identity over time when cultured in 2D conditions in vitro, including upregulation of AT1-associated markers (26). We therefore confirmed that iAT2s in ALI cultures maintain or upregulate their AT2 transcriptomic program at the single-cell level and, at the protein level, maintain expression of the key lineage markers SFTPC and NKX2.1. Essential functions of AT2s include the production of surfactant lipids and proteins, which are stored in organelles known as lamellar bodies, and maintenance of barrier integrity between the ventilated alveolar space and the perfused pulmonary vasculature. We found that iAT2s at ALI generate lamellar bodies as they do in 3D culture (14), consistent with our prior observation that iAT2s at ALI secrete the functional form of secreted surfactant, tubular myelin (18). We also observed that iAT2s cultured in ALI generate tight junctions with an associated increase in TEER over time in culture, in contrast with iAT2s in 2D submerged culture. Together, these findings demonstrate that iAT2s at ALI recapitulate critical AT2 functional characteristics.

To further characterize the effects of ALI culture, we applied scRNA-Seq to compare the global transcriptome of iAT2s cultured in ALI directly to other culture formats, including 2D submerged and 3D alveolosphere culture. We found that sets of genes associated with primary AT2 differentiation and maturation (15) were upregulated in iAT2s cultured at ALI compared with those cultured in 3D or in 2D submerged conditions. Concurrently, we observed that iAT2s at ALI were less likely to be actively cell cycling compared with those in 3D through a combination of transcriptomic cell cycle phase predictions and EdU incorporation assays. Our results suggest an inverse relationship between maturation and cell proliferation, which promotes maturation of iAT2s with decreased cell cycling when cultured at ALI.

Our group has previously compared the 3D iAT2 transcriptome to primary fetal and adult AT2s (14, 15, 21). In this study, we extended this analysis to iAT2s cultured at ALI with additional comparisons with cultured pediatric and adult AT2s. Acknowledging the potential contributions of batch effects across sequencing runs, we directly compared gene expression levels only between samples sequenced together and correlated on larger gene expression signatures between samples from different sequencing experiments. We found that iAT2s in ALI culture are transcriptomically more similar to primary postnatal AT2s than to 3D alveolospheres or primary fetal comparators. As is true for 3D iAT2s (14), the main drivers of differential gene expression between primary AT2s and iAT2s in ALI culture were attributable to exposure to immune stimuli. Primary sorted adult AT2s expressed high levels of IFN response and Inflammatory Response transcripts, in contrast to very low or no expression in iAT2s cultured in vitro in either 3D or ALI format.

The interface of alveolar epithelial cells with the outside environment is central to the pathogenesis of a variety of lung diseases. Among these, inhaled pollutants such as cigarette smoke have particular longstanding worldwide significance. In contrast to the alveolar epithelial response, the human airway epithelial response to cigarette smoke has been well studied (27–29). Whole transcriptome analysis of bronchial brushings from subjects who currently smoke compared with those who never smoked has shown a distinct transcriptomic signature, including enrichment of antioxidant and xenobiotic metabolism pathway transcripts (29). This airway transcriptional signature of in vivo chronic smoke exposure is largely concordant with the transcriptional signature of in vitro cultured primary bronchial epithelial cells that have been acutely and intensely exposed to whole cigarette smoke at ALI (27, 28). The analogous primary model system for the alveolar epithelium, however, is much less developed compared with the primary bronchial epithelial cell–based system. Cancer and immortalized cell lines have been used in alveolar epithelial ALI cultures (30, 31). Aside from studies of whole lung tissue (32), there are no published transcriptomic data sets to our knowledge profiling the response of AT2s from human subjects who smoke compared with those who have never smoked, and primary human AT2s have not been characterized in terms of their response to cigarette smoke in vitro. Applying our model, we found that iAT2s in ALI culture upregulate reactive oxygen species and xenobiotic metabolism responsive genes in response to cigarette smoke exposure. This response is similar to the acute response to cigarette smoke observed in airway epithelial cells exposed to cigarette smoke in vitro (27, 28), suggesting a common epithelial smoke–responsive transcriptomic program.

Given the high and increasing prevalence of electronic cigarette use among school-aged children in the United States (33), their marketing as a safer alternative to cigarettes, and a lingering question in the field as to whether the effects of electronic cigarette vapor are diminished in relation to or differ from those of combustible cigarette smoke (34, 35), we directly compared the response of iAT2s with combustible cigarette smoke and electronic cigarette vapor. We observed a similar induction of a set of smoke-responsive transcripts both in magnitude and timing following exposure to electronic cigarette vapor, with a peak transcriptomic response occurring 2–6 hours after exposure. This finding reveals a degree of concordance between the iAT2 response to these exposures despite differences in their composition and suggests the need for further studies to fully characterize potential toxic effects of electronic cigarette vapor in human alveolar cells. In summary, these studies demonstrate the utility of the iAT2 ALI system for the direct comparison of the cellular response to airborne stimuli and, in particular, raise the possibility that electronic cigarette vapor and cigarette smoke have similar acute effects on alveolar epithelial cells. Future studies should comprehensively evaluate the alveolar epithelial response to electronic cigarette vapor exposure alone or in combination with cigarette smoke, a common use pattern observed clinically (36).

## Methods

### iAT2 ALI culture.

iAT2s in 3D spheres were generated as previously described (14, 21) and plated in Growth Factor Reduced Matrigel (Corning) droplets at a density of 400 cells/μL. Matrigel droplets were dissolved in 2 mg/mL dispase (MilliporeSigma), and alveolospheres were dissociated in 0.05% trypsin (Thermo Fisher Scientific) to generate a single-cell suspension. Transwell inserts (6.5 mm; Corning) were coated with dilute hESC-Qualified Matrigel (Corning) according to the manufacturer instructions. Single-cell iAT2s were plated on Transwells at a density of 520,000 live cells/cm^2^ in 100 μL of CK+DCI+Y (3 μM CHIR99021, 10 ng/mL KGF, 50 nM dexamethasone, 0.1 mM cAMP, 0.1 mM IBMX, 10 μM Rho-associated kinase inhibitor [MilliporeSigma, Y-27632]). CK+DCI+Y (500 μL) was added to the basolateral compartment. Twenty-four hours after plating, basolateral medium was refreshed with CK+DCI+Y. Forty-eight hours after plating, apical medium was aspirated to initiate ALI culture. Seventy-two hours after plating, basolateral medium was changed to CK+DCI to remove the Rho-associated kinase inhibitor. Basolateral medium was changed 3 times per week thereafter.

### iAT2 ALI immunofluorescence staining.

iAT2 ALI cultures were fixed with 4% paraformaldehyde (Electron Microscopy Sciences [EMS], 19208) for 15 minutes. Cultures were washed, permeabilized using 0.25% Triton X-100 (MilliporeSigma, 9002-93-1) for 30 minutes at room temperature (RT), and then blocked with 2.5% normal donkey serum (MilliporeSigma, D9663) for a total of 1 hour at RT. Cells were incubated with anti–pro-SFTPC (Santa Cruz Biotechnology Inc., sc-518029, 1:500) primary antibody overnight at 4°C. Cells were washed and incubated with secondary antibody conjugated to Alexa Fluor 488 (Jackson ImmunoResearch, 711-545-150, 1:500) for 2 hours at RT. Nuclei were counterstained with Hoechst 33342 dye (Thermo Fisher Scientific, H3570, 1:500). Transwell membranes were excised with a razor blade and mounted on glass slides using Prolong Diamond Anti-Fade Mounting Reagent (Thermo Fisher Scientific, P36931) and visualized with a Zeiss confocal microscope.

### Intracellular flow cytometry for NKX2-1.

iAT2 ALIs were dissociated using Accutase (MilliporeSigma, A6964) according to the manufacturer instructions. Cells were evaluated for intracellular NKX2-1 protein by flow cytometry as previously described (23).

### scRNA-Seq.

For scRNA-Seq, SPC2-ST-B2 (hereafter referred to as SPC2) (15) iAT2s were cultured as 3D spheres, on Transwells in 2D submerged conditions, or at ALI for a total of 10 days after passaging. iAT2s in ALI culture were exposed to cigarette smoke 12 hours prior to cell collection (Smoke) or air (ALI). On day 10, and at 12 hours after cigarette smoke exposure, cultures were dissociated to single cells, and live cells were sorted on calcein blue on a Mo-Flo Astrios Cell Sorter.

Capture and library preparation was performed using a 10X Chromium system. Libraries were sequenced using a NextSeq 500. Fastq files were generated and counts extracted from each of the 4 samples using bcl2fastq v.2.2 and cellranger v.3.0.2. The expression libraries were mapped to the human genome (GRCh38) with GFP and tdTomato reporters. The 3D sample generated 2363 cells at a depth of 45,726 reads/cell. The 2D sample generated 1135 cells at a depth of 96,307 reads/cell. The ALI sample generated 1539 cells at a depth of 72,633 reads/cell. The smoke-exposed sample generated 981 cells at a depth of 96,022 reads/cell. We used Seurat v.3 (37) to further process the data. Cells with more than 20% of reads mapping to mitochondrial genes and doublet cells were filtered out. The 4 samples were merged and then normalized using SCTransform with cell degradation effects regressed out. The dimensionality reductions PCA and Uniform Manifold Approximation and Projection (UMAP) were used to represent the gene expression, and the Louvain algorithm was used for clustering. Differential expression tests were then done with the MAST algorithm (38). All raw data files can be downloaded from Gene Expression Omnibus (GEO). GSE184142 contains this scRNA-Seq data set and is part of Super Series GSE184144, which contains both sequencing experiments described in this manuscript.

### TEER.

TEER was measured using a Millicell ERS-2 Voltohmmeter (MilliporeSigma, MERS00002). Electrodes were sterilized in 70% ethanol and conditioned in cell culture medium before measurements were taken. Cell culture medium (200 μL) was added to the apical chamber of Transwell culture inserts. Readings were taken at 3 locations in every well, as well as a blank Transwell coated with dilute hESC-Qualified Matrigel but without iAT2s. The difference between the mean well reading and the mean blank reading was multiplied by tissue culture growth area (0.33 cm^2^ for a 6.5 mm diameter Transwell) to calculate TEER for each well. 

### iAT2 ALI TEM.

iAT2 ALI cultures on Transwell inserts fixed in 2% glutaraldehyde + 1% paraformaldehyde in 0.1M cacodylate buffer at pH 7.4 for 1 hour at RT. They were then washed in cacodylate buffer + 2% sucrose and postfixed in 1.5% osmium tetroxide (Polysciences) overnight at 4°C. The membrane was excised from the insert, block stained in 1.5% uranyl acetate (EMS) for 1 hour at RT. The samples were dehydrated quickly through acetone on ice, from 70% to 80% to 90%. The samples were then incubated 2 times in 100% acetone at RT for 10 minutes each, and in propylene oxide at RT for 15 minutes each. Finally, the samples were changed into EMbed 812 (EMS), left for 2 hours at RT, changed into fresh EMbed 812, and left overnight at RT, after which they were embedded in fresh EMbed 812 and polymerized overnight at 60°C. Embedded samples were thin sectioned (70 nm), and grids were stained in 4% aqueous uranyl acetate for 10 minutes at RT, followed by lead citrate for 10 minutes at RT. Electron microscopy was performed on a Philips CM12 EM operated at 100 kV, and images were recorded on a TVIPS F216 CMOS camera with a pixel size of 2.89 nm per pixel. 

### qPCR.

iAT2s were collected in Qiazol (Qiagen), and RNA was extracted with the RNeasy mini kit (Qiagen). MultiScribe Reverse Transcriptase (Applied Biosystems) was used to generate complementary DNA (cDNA). Using a QuantStudio instrument (Applied Biosystems) and predesigned TaqMan probes (Applied Biosystems), 384-well PCR was run for 40 cycles. Relative transcript expression was calculated as fold change over the control group indicated on the *y* axis of each plot using the 2^–ΔΔCt^ method (39) and *18S* as the internal control.

### EdU flow cytometry assay.

EdU incorporation was assessed using the Click-iT Plus EdU Alexa Fluor 647 Flow Cytometry Assay Kit (Thermo Fisher Scientific). EdU (10 μM) was added to the basolateral compartment of iAT2s on day 9 after passaging to cell culture inserts. On day 10, after 24 hours of EdU incorporation, iAT2s were collected with Accutase (MilliporeSigma). iAT2s were fixed and assayed for EdU according to the manufacturer’s instructions. Flow cytometry was performed on a Stratedigm (S1000EXI) cytometer and analyzed using FlowJo (BD Biosciences).

### Primary AT2 isolation and culture.

Human distal lung samples were obtained from a 13-month-old male donor (pediatric lung cells) and a 32-year-old male donor with no smoking history (adult lung cells). Both donors died of traumatic injuries, and their lungs were deemed unsuitable for transplantation. Distal human lung samples were treated with Liberase (MilliporeSigma) for 60 minutes at 37°C and disaggregated by pipetting and filtered through 40 μm cell strainers (Falcon) twice. Cells were stained for EpCAM (BioLegend), CD31 (BioLegend), CD45 (BioLegend), NGFR (BioLegend), and HTII-280 (Terrace Biotech). EPCAM^+^CD45^–^CD31^–^NGFR^–^HTII-280^+^ cells (AT2s) were sorted on a BD Fusion FACS instrument.

After sorting, 5000 AT2s and 25000 MRC-5 fibroblasts (ATCC) in Growth Factor Reduced Matrigel (Corning) were seeded per 6.5 mm diameter Transwell cell culture inserts (Corning) with CK+DCI medium (14, 21) in the basolateral compartment.

### Bulk RNA-Seq.

For bulk RNA-Seq, SPC2 (15) iAT2s were cultured as 3D spheres or at ALI (*n* = 3 per condition) for a total of 181 days of differentiation, including 10 days after passaging. On day 10, Matrigel of 3D cultures was dissolved in dispase (Thermo Fisher Scientific) for 30 minutes at 37°C. Spheres were pelleted and lysed in Qiazol (Qiagen). ALI cultures were lysed in Qiazol (Qiagen) added directly to cell culture inserts. 

Primary AT2s were cultured in Growth Factor Reduced Matrigel for 106 days after HTII-280 sorting. Matrigel was dissolved in dispase (Thermo Fisher Scientific) for 30 minutes at 37°C, and alveolospheres with MRC5s were washed once with PBS. Mixtures of alveolospheres and free MRC5s in PBS were allowed to separated by gravity for 5 minutes on ice. Supernatants enriched for MRC5s were removed, and alveolospheres mixed with trace MRC5s were lysed in TRIzol (Invitrogen). 

RNA was isolated from all samples in a single batch using the RNeasy Mini kit (Qiagen), including on-column DNA digestion with the RNase-free DNAse Set (Qiagen). The mRNA was isolated from each sample using magnetic bead-based poly(A) selection, fragmented, and reverse transcribed to cDNA fragments. cDNA was end-repaired, ligated to Illumina sequencing adapters, and PCR-amplified to generate cDNA libraries. Pooled libraries were sequenced using a NextSeq500 (Illumina). 

The quality of the raw data was assessed using FastQC v.0.11.7 (40). The sequence reads were aligned to the human reference genome (GRCh38), the SARS-CoV-2 virus reference (NC_045512), and the tdTomato marker using STAR v.2.6.0c (41). Counts per gene were summarized using the featureCounts function from the subread package v.1.6.2 (42).

All raw data files can be downloaded from GEO. GSE184143 contains this RNA-Seq data set and is part of Super Series GSE184144, which contains both sequencing experiments described in this manuscript.

### Deconvolution of primary cultured AT2 expression profiles.

In order to impute the expression profile of each cell type in samples with cocultures of AT2s and MRC5 fibroblasts, we used an analytical tool called CIBERSORTx (43). In order to avoid limitations in the number of genes imputed (only 1000 genes or fewer in the browser version due to computationally intensive requirements), we used the full-transcriptome version available as a Docker container. We first generated a signature matrix using a scRNA-Seq data set (unpublished) of primary AT2s cocultured with MRC5s using the same culture method described here (prefilter: 400 markers ranked by log fold change in each cell type; parameters for the signature construction in CIBERSORTx: min differentially expressed gene [DEG] = 300, max DEG = 500, *q* value = 0.01, quantile normalization enabled, κ = 999, min. expression = 1) (Supplemental Table 1). The full signature was then used to estimate the percentage of each cell type in each library and to impute the expression profile of all genes in the imputed cell types per library (High-Resolution Mode) (Supplemental Table 2). Deconvolved expression profiles for primary adult and pediatric cultured AT2s were used for downstream analysis and are hereafter referred to as pediatric cultured AT2 and adult cultured AT2 (*n* = 3 per group).

### Differential expression analysis.

Expression profiles of undifferentiated PSCs (day 0), NKX2.1+ PSC-derived progenitors after 15 days of directed differentiation (day 15), SFTPC-tdTomato–sorted iAT2s cultured as spheres after 35 days of directed differentiation (day 35), distal epithelial cells from HFLs at 16–17.5 weeks of gestation (early HFL) and 20–21 weeks (late HFL), and adult HTII-280–sorted (24) primary AT2 controls (adult sorted AT2) were previously generated by RNA-Seq (*n* = 3 per group) (14, 15). These expression profiles were merged with newly generated iAT2 3D and ALI and deconvolved adult and pediatric cultured AT2 data sets.

After the summarization of counts per gene using featureCounts, the edgeR package v.3.25.10 (44) was used to import, organize, and filter the counts, and the matrix of counts per gene per sample was then analyzed using the limma/voom normalization method (45). Genes were filtered based on the standard edgeR filtration method using the default parameters for the “filterByExpr” function.

After exploratory data analysis (Glimma v. 1.11.1), contrasts for differential expression testing were done to compare adult sorted AT2 versus adult cultured AT2, adult sorted AT2 versus iAT2 ALI, and adult cultured AT2 versus iAT2 ALI. The limma package v.3.42.2 (45) with its voom method — namely, linear modeling and empirical Bayes moderation — was used to test differential expression (moderate *t* test). *P* values were adjusted for multiple testing using Benjamini-Hochberg correction FDR *P* value. Differentially expressed genes for each comparison were visualized using Glimma v. 1.11.1 (46), and FDR < 0.05 was set as the threshold for determining significant differential gene expression. Functional predictions were performed using the fgsea v.1.12.0 package (47) and for gene set analysis.

### Primary sample correlation metrics.

In order to quantify the relative similarity between the iAT2s in different culture conditions to the fresh AT2s and to undifferentiated cells, we reanalyzed the deconvoluted iAT2 samples together with the fresh primary and undifferentiated samples to be used as benchmarks. All samples were normalized together from raw counts, and we followed the standard procedure outlined above for RNA-Seq data analysis. Then, we selected the informative features to be used for the correlation: for an independent selection, we took the top 50 markers of each epithelial cell type in the single-cell IPF lung atlas (25). This includes the following categories: MUC5AC^+^ High, Basal, Proliferating Epithelial Cells, Differentiating Ciliated, Ciliated, SCGB3A2^+^SCGB1A1^+^, SCGB3A2^+^, MUC5B^+^, KRT5^–^KRT17^+^, Transitional AT2, AT2, and AT1. Then, we calculated the Pearson correlation coefficient between the normalized expression (counts per million) of the selected features in each sample. The resulting coefficients are visualized in heatmaps (Figure 3C and Supplemental Figure 4).

### In vitro combustible cigarette smoke exposure.

iAT2s cultured at ALI were exposed to gas-phase combustible cigarette smoke in a Vitrocell VC1 in vitro smoke exposure system (Vitrocell Systems). University of Kentucky 3R4F reference cigarettes were preconditioned for at least 48 hours prior to smoke exposure in accordance with ISO 3402, described in ref. 48. Cigarettes were conditioned in a temperature and humidity-controlled chamber at 22°C ± 1°C and a relative humidity of 60% ± 2%. The smoke exposure protocol followed ISO 3308, described in ref. 49. Every 60 seconds, 35 mL puffs were drawn over a duration of 2 seconds. For each smoke exposure, 4 cigarettes were used with 8 puffs drawn per cigarette, for a total of 32 puffs per exposure. Smoke was diluted with room air humidified to > 90% in order to expose ALI cultures to 5% (v/v) cigarette smoke.

### In vitro electronic cigarette vapor exposure.

iAT2s at ALI were exposed to electronic cigarette vapor using the Vitrocell VC1 in vitro smoke exposure system (Vitrocell Systems). A custom adaptor was 3D-printed so that an electronic cigarette could be placed in the VC1 cigarette holder. A fully charged JUUL brand electronic cigarette was used with 5% nicotine “Virginia Tobacco” flavored JUULpods. Vapor was drawn in accordance with ISO 3308 (49), as described for combustible cigarette smoke exposures. For each electronic cigarette vapor exposure, a total of 32 puffs were drawn from the JUULpod through the JUUL device. Vapor was diluted with room air humidified to > 90% in order to expose ALI cultures to 5% (v/v) electronic cigarette vapor.

### IL-8 ELISA.

IL-8 ELISA was performed using the human IL-8/CXCL8 DuoSet ELISA kit (R&D Systems) according to the manufacturer’s instructions.

### Statistics.

Unpaired, 2-tailed Student’s *t* tests were used to compare quantitative measures in 2 groups of 3 or more samples. One-way ANOVA with multiple comparisons was used to compare 3 or more groups. Replicates used for each experiment are outlined in figure legends. The *P* value threshold to determine significance was set at *P* = 0.05.

### Study approval.

iPSC studies in this manuscript were performed with a previously published iPSC line, SPC2 (15, 22). Primary samples obtained to generate the parental line were obtained with written, informed consent. All iPSC-directed differentiation experiments were performed with the approval of the IRB of Boston University (protocol no. H33122). Further details of iPSC derivation, characterization, and culture are available for free download at https://www.bu.edu/dbin/stemcells/protocols.php

## Author contributions

KMA, AAW, and DNK conceived of the study and designed experiments. JSDA, AR, CGDA, RJ, and CFK provided resources and designed experiments. KMA, JSDA, KDA, AR, CGDA, JH, OTH, RBW, EB, AH, and IF performed experiments. KMA, JLV, and CVM analyzed sequencing data. KMA and AAW drafted the manuscript. All authors edited and approved the final manuscript. AAW supervised the study.

## Supplementary Material

Supplemental data

Supplemental tables 1-4

## Version 1 03/22/2022

Electronic publication

## Version 2 03/25/2022

The supplemental material was added
