# Supplementary material for: Air-liquid interface culture promotes maturation and allows environmental exposure of pluripotent stem cell–derived alveolar epithelium
Source: JCI Insight. 2022 Mar 22;7(6):e155589. doi: 10.1172/jci.insight.155589 (PMC8986076; doi:10.1172/jci.insight.155589)
Supplement: Supplemental data [file jciinsight-7-155589-s272.pdf]

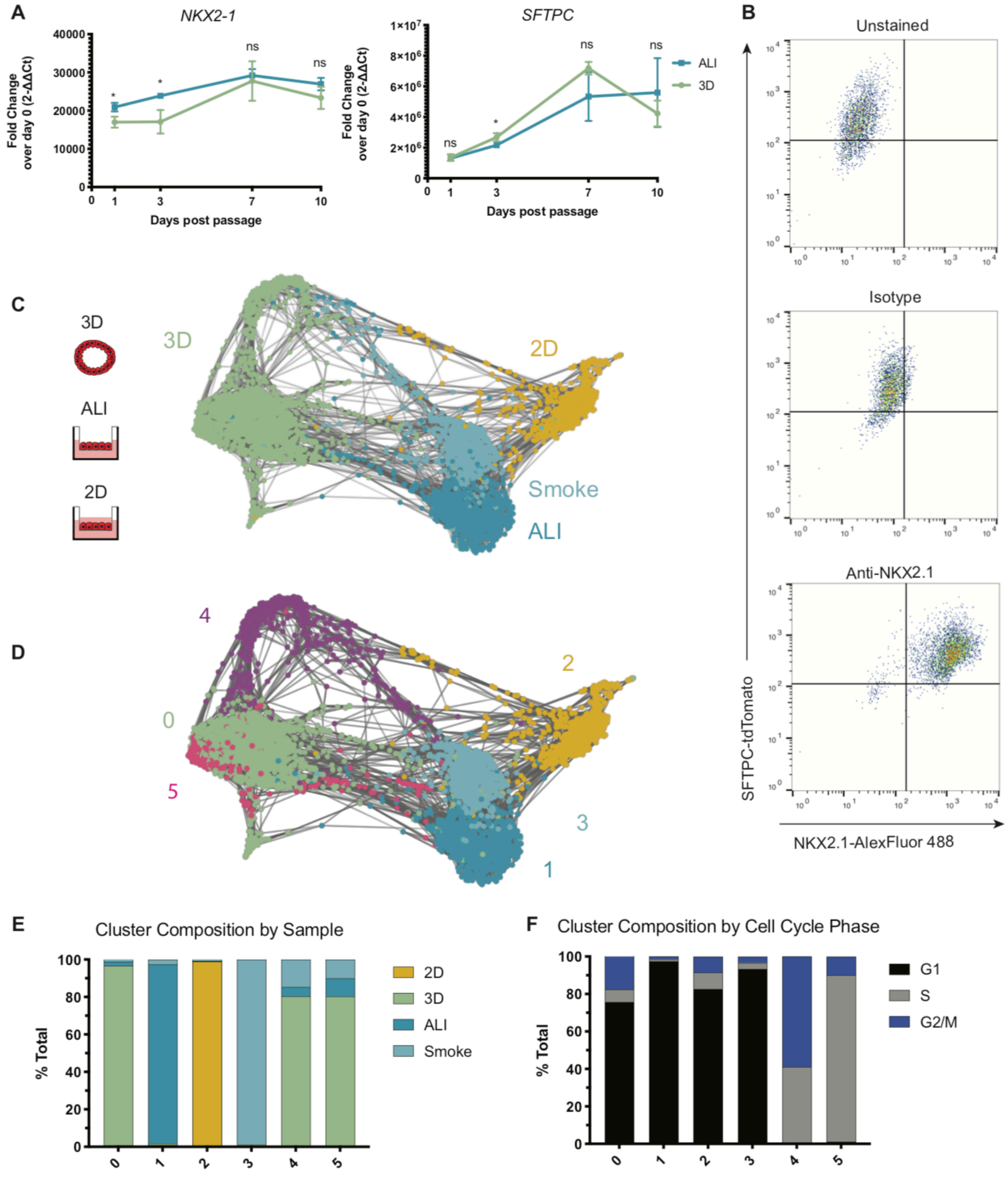


**Supplemental Figure S1.**

(A) RT-qPCR of *NKX2-1* and *SFTPC* expression at 1, 3, 7, and 10 days post passage in iAT2s cultured at 3D or ALI (mean ± standard deviation, unpaired two-tailed Student's t test, n=3)

(B) Representative flow cytometry plots of iAT2s with SFTPC$^{tdTomato}$ reporter cultured in ALI and stained for NKX2.1, table of percent NKX2.1+, tdTomato+, and NKX2.1/tdTomato double + cells per replicate (n=3).

(C) SPRING plot of iAT2s cultured in 3D, 2D, or ALI and profiled by scRNA-Seq 12 hours after cigarette smoke exposure (Smoke) or air exposure (ALI), colored by condition.

(D) SPRING plot colored by Louvain cluster (res=0.25).

(E) Sample contribution to each Louvain cluster (res=0.25).

(F) Transcriptomically predicted cell cycle phase of each Louvain cluster (res=0.25).

*Mean ± standard deviation. *p≤0.05, **p≤0.01, ***p≤0.001, ****p≤0.0001.*

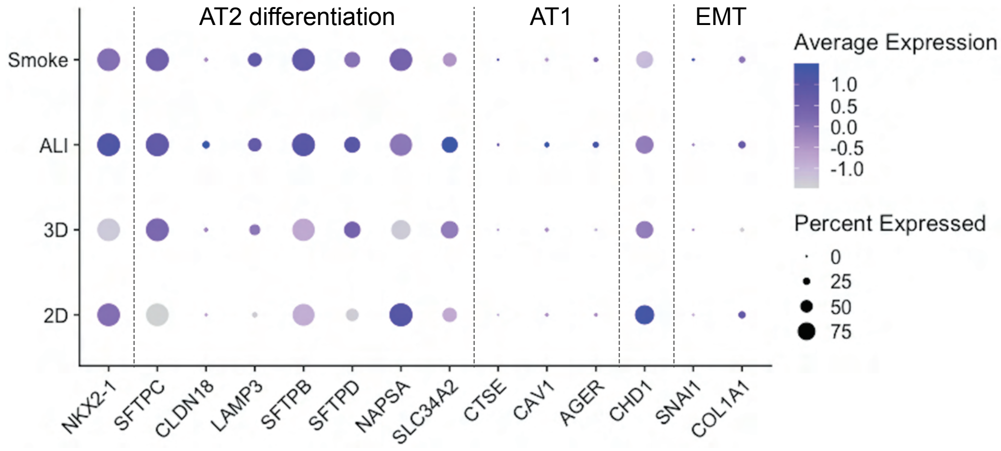


**Supplemental Figure S2.** Dot plot of gene expression in iAT2 cultured in 3D, ALI, and 2D profiled on day 10 by scRNA-Seq.

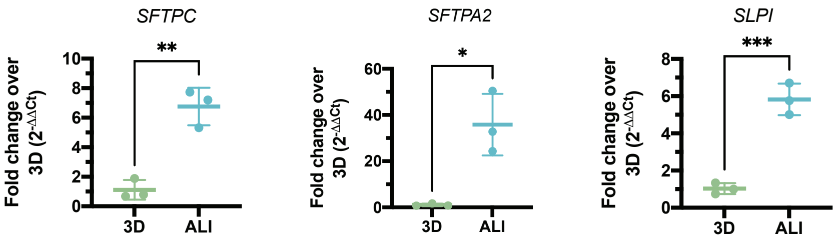


**Supplemental Figure S3.** RT-qPCR of AT2 gene expression *SFTPC, SFTPA2, SLPI* in iAT2s generated from the iPSC line BU3 cultured in 3D versus ALI. (mean ± standard deviation, unpaired two-tailed Student's t test, n=3)
*Mean ± standard deviation. *p≤0.05, **p≤0.01, ***p≤0.001.*

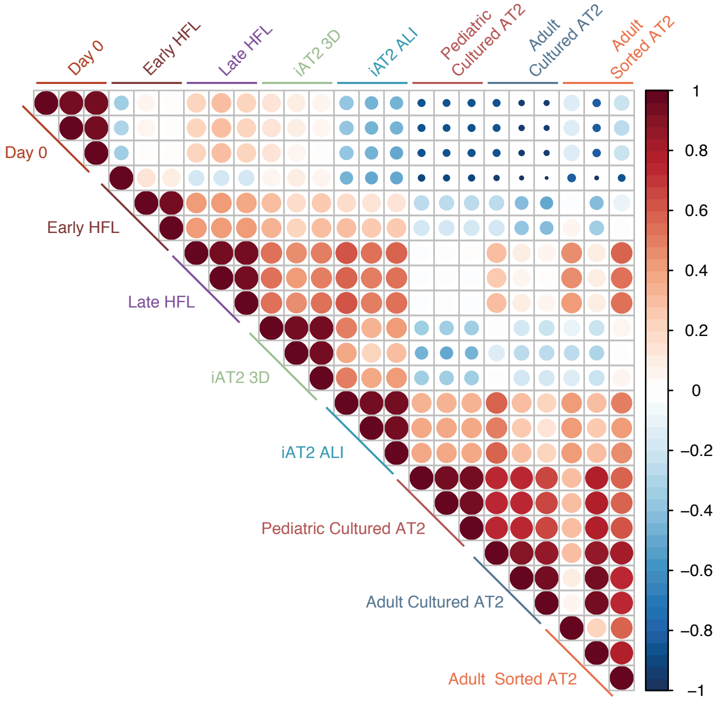


**Supplemental Figure S4.** Heatmap of Pearson correlation coefficients calculated between each sample based on normalized expression of human lung epithelial genes from an independent dataset.

**TABLES IN SUPPLEMENTAL DATA:**

**Table 1.** CIBERSORT signature matrix containing relative expression values of each gene (rows) in fibroblasts or AT2s (columns).

**Table 2.** Table of proportional contribution in each sample attributable to fibroblast or AT transcripts (CIBERSORT weights).

**Table 3.** Pearson correlation coefficients, individual comparisons, visualized as heat map in Supplemental Figure S3 and as group averages in Figure 3C.

**Table 4.** Hallmark gene sets enriched in Adult Sorted AT2 vs iAT2 ALI (FDR<0.05, ranked by direction of differential expression and P value).
